# Exceptional and Durable Complete Response to Ado-Trastuzumab Emtansine in HER2-Positive Metastatic Breast Cancer: A Case Report

**DOI:** 10.7759/cureus.102669

**Published:** 2026-01-30

**Authors:** Yuko Tashima, Yurie Araki, Shinichi Araki, Yuki Tahara, Fumihiro Tanaka

**Affiliations:** 1 Second Department of Surgery, School of Medicine, University of Occupational and Environmental Health, Kitakyushu, JPN

**Keywords:** ado-trastuzumab emtansine, anti-her2 therapy, case report, complete response, durable response, exceptional responder, her2-positive metastatic breast cancer, long-term survival

## Abstract

Ado-trastuzumab emtansine (T-DM1) is an established treatment for patients with human epidermal growth factor receptor 2 (HER2)-positive metastatic breast cancer who have previously received trastuzumab-based therapy. However, complete and durable responses to T-DM1 are uncommon, and long-term complete remission has rarely been reported.

We report the case of a 72-year-old woman with HER2-positive metastatic breast cancer who achieved a sustained complete response following T-DM1 therapy. The patient was initially diagnosed with left breast invasive ductal carcinoma with axillary and abdominal lymph node metastases. She received first-line chemotherapy with trastuzumab, pertuzumab, and docetaxel, followed by maintenance anti-HER2 therapy and surgery for local disease control. During maintenance therapy, chest wall recurrence with rib and mediastinal lymph node involvement was detected. Re-biopsy confirmed persistent HER2 overexpression. After progression on endocrine therapy, T-DM1 was initiated. Marked tumor regression was observed on CT imaging three months after initiation of T-DM1, and complete radiological remission has been maintained for more than five years with continuous T-DM1 treatment. Adverse events were limited to mild liver dysfunction, and no clinically significant thrombocytopenia occurred.

This case may represent an exceptional response to T-DM1 and highlights the potential for durable complete remission in selected patients with HER2-positive metastatic breast cancer. Further investigation is warranted to identify predictive factors for exceptional responses and to determine optimal treatment duration.

## Introduction

Human epidermal growth factor receptor 2 (HER2)-positive breast cancer accounts for approximately 15% to 20% of all breast cancer cases and has historically been associated with aggressive tumor behavior and poor prognosis. Early studies demonstrated that amplification of the HER2/neu oncogene correlated with increased rates of relapse and reduced survival in patients with breast cancer, establishing HER2 as a key prognostic and therapeutic target [1].

The introduction of HER2-targeted therapies, beginning with trastuzumab, has dramatically improved clinical outcomes for patients with HER2-positive breast cancer. Subsequent development of combination anti-HER2 strategies and novel agents has further transformed the treatment landscape, leading to prolonged survival even in the metastatic setting [2].

Ado-trastuzumab emtansine (T-DM1) is an antibody-drug conjugate composed of trastuzumab linked to the cytotoxic agent emtansine (DM1). This structure enables selective delivery of the cytotoxic payload to HER2-overexpressing tumor cells while preserving the anti-HER2 activity of trastuzumab. The pivotal EMILIA and TH3RESA trials established T-DM1 as an effective treatment option for patients with HER2-positive metastatic breast cancer previously treated with trastuzumab and taxanes [3,4].

Despite these advances, complete and durable responses to T-DM1 remain uncommon in routine clinical practice. In pivotal clinical trials such as EMILIA, complete responses to T-DM1 were rare, occurring in approximately 1% of patients, and long-term durability of such responses beyond several years has only rarely been described in the literature [4]. Here, we report a rare case of sustained complete response lasting more than five years following T-DM1 therapy in a patient with HER2-positive metastatic breast cancer.

## Case presentation

**Figure 1:**
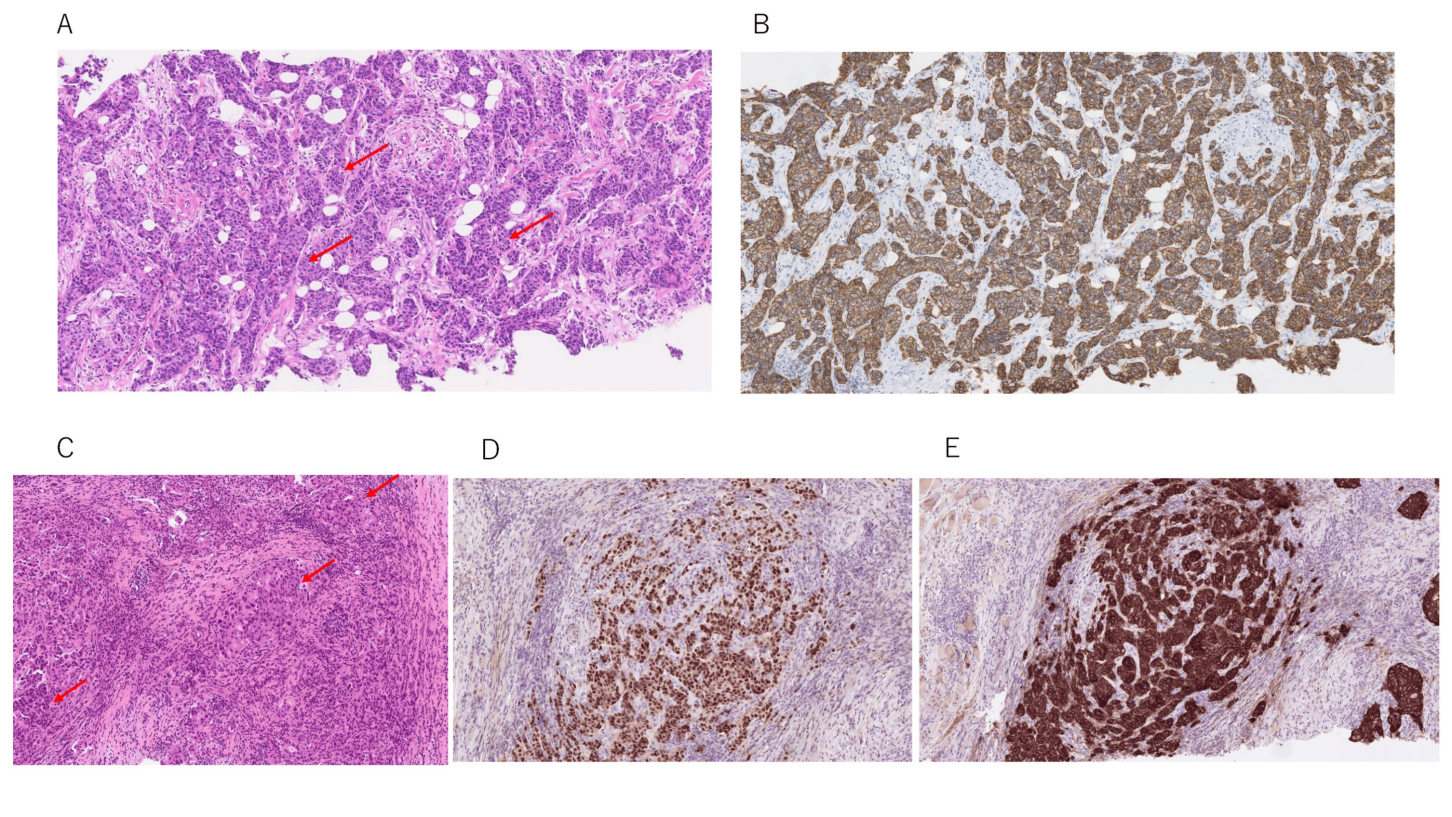
Histopathological findings at initial diagnosis and chest wall recurrence Red arrows indicate representative cancer cells. (A) Hematoxylin and eosin (H&E) staining of the initial needle biopsy specimen showing invasive ductal carcinoma (original magnification: ×100). (B) Immunohistochemical staining of the initial biopsy specimen demonstrating strong human epidermal growth factor receptor 2 (HER2) overexpression (score 3+) (original magnification: ×100). (C) H&E staining of the chest wall recurrence obtained by re-biopsy, confirming recurrent carcinoma (original magnification: ×100). (D) Immunohistochemical staining of the re-biopsy specimen from the chest wall recurrence showing persistent strong estrogen receptor (ER)–positive tumor cells (original magnification: ×100). (E) Immunohistochemical staining of the re-biopsy specimen from the chest wall recurrence showing persistent strong HER2 overexpression (score 3+) (original magnification: ×100).

A 72-year-old woman was diagnosed with left breast cancer in March 2016. Histopathological examination of a needle biopsy specimen revealed invasive ductal carcinoma of the scirrhous type. Immunohistochemical analysis demonstrated estrogen receptor (ER) positivity (60%), progesterone receptor negativity, and strong HER2 overexpression (3+ by immunohistochemistry), with a Ki-67 labeling index of 42.7% (Figure 1).

Initial staging showed a primary tumor measuring 26 mm with ipsilateral axillary lymph node involvement and abdominal lymph node metastasis, consistent with cT2N1M1 stage IV disease.

From April 2016, the patient received first-line systemic therapy with trastuzumab, pertuzumab, and docetaxel. After six cycles, docetaxel was discontinued due to adverse effects, and maintenance therapy with trastuzumab and pertuzumab was continued. In June 2017, surgical resection was performed for local disease control. Under maintenance anti-HER2 therapy, the patient remained clinically stable, and metastatic lesions, including abdominal lymph node metastases, were no longer radiologically detectable.

**Figure 2:**
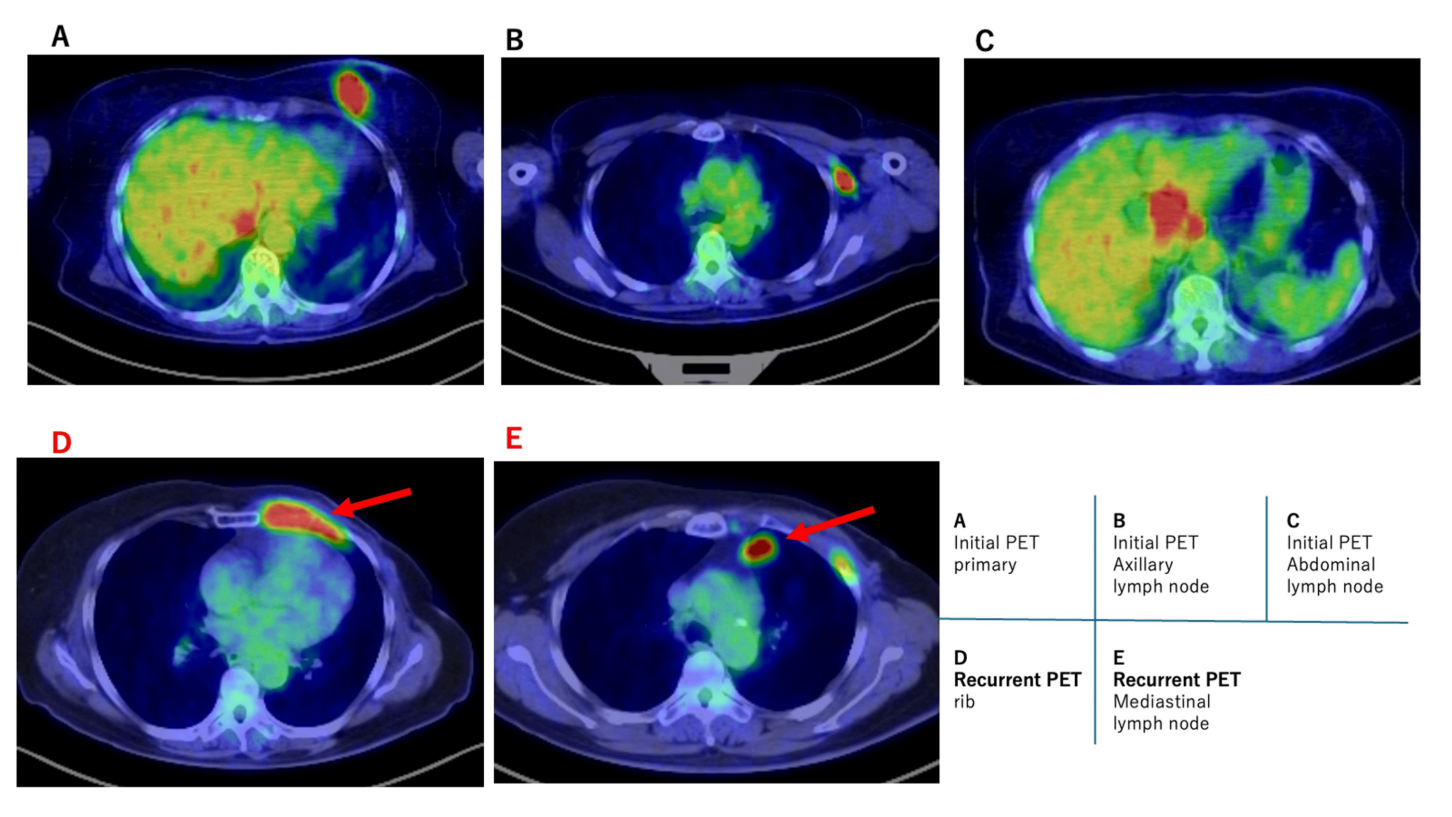
Positron emission tomography (PET) findings at initial diagnosis and recurrence Initial PET images demonstrate increased uptake in the primary breast tumor (A), axillary lymph node (B), and abdominal lymph node (C). PET images obtained at recurrence show abnormal uptake in the left third rib (D) and mediastinal lymph node (E) (arrows).

In July 2020, the patient developed pain in the left anterior chest wall. Contrast-enhanced computed tomography and positron emission tomography revealed abnormal uptake in the left third rib and enlargement of mediastinal lymph nodes (Figure 2). Re-biopsy of the chest wall lesion confirmed recurrent carcinoma with persistent ER positivity (>95%), HER2 overexpression (3+), and a Ki-67 index of 20%. Endocrine therapy was attempted but showed no clinical benefit. Comprehensive genomic profiling, including next-generation sequencing and circulating tumor DNA analysis, was not performed in this case.

In December 2020, treatment with T-DM1 was initiated at a dose of 3.6 mg/kg.

**Figure 3:**
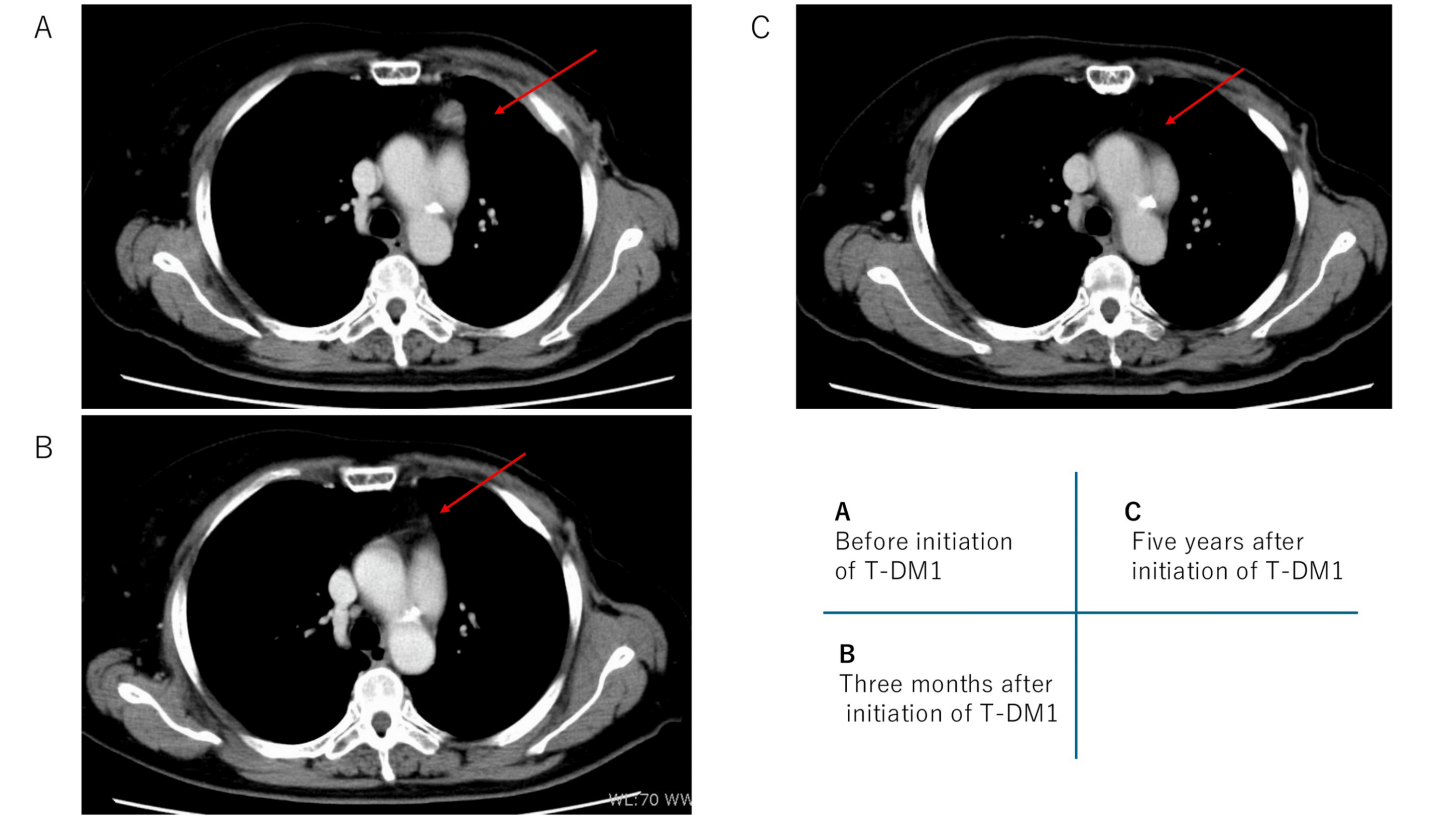
Serial follow-up computed tomography images demonstrating sustained complete response of mediastinal lymph nodes following ado-trastuzumab emtansine (T-DM1) therapy (A) CT image obtained prior to initiation of T-DM1, showing enlarged mediastinal lymph nodes (red arrow). (B) CT image obtained three months after initiation of T-DM1, demonstrating complete disappearance of the mediastinal lymph nodes, consistent with a complete response. (C) CT image obtained five years after initiation of T-DM1, showing sustained complete response with no evidence of recurrent mediastinal lymphadenopathy.

Follow-up CT performed approximately three months later demonstrated resolution of mediastinal lymph node metastases (Figure 3).

**Figure 4:**
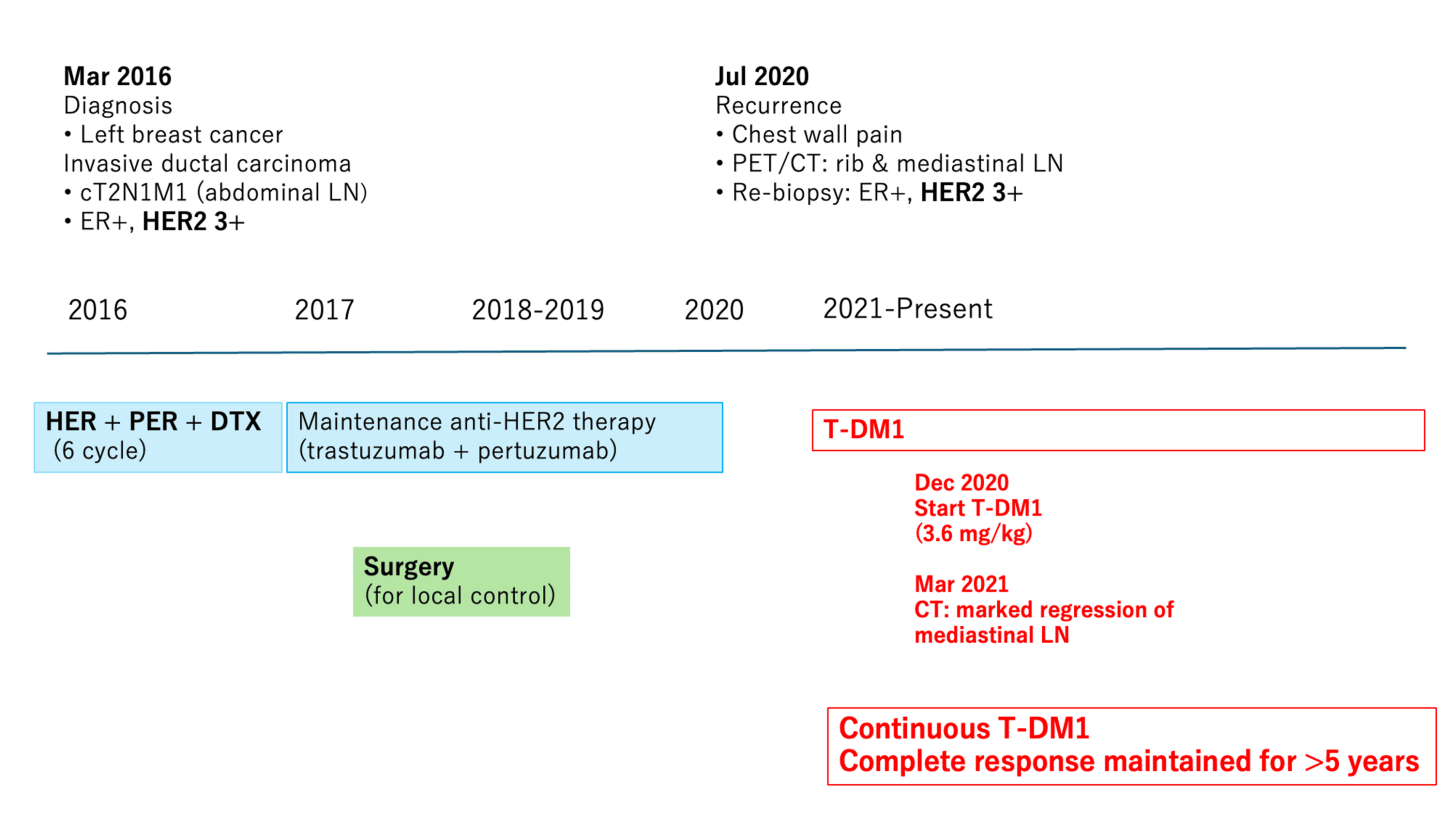
Treatment timeline Timeline summarizing the clinical course and treatments administered from initial diagnosis to long-term follow-up. After first-line chemotherapy with HER, PER, and DTX, maintenance therapy with HER and PER was continued. Chest wall recurrence occurred during maintenance therapy, and subsequent treatment with ado-trastuzumab emtansine (T-DM1) resulted in sustained complete response for more than five years. HER-2: human epidermal growth factor receptor 2; HER: trastuzumab; PER: pertuzumab; DTX: docetaxel; LN: lymph node

Based on the disappearance of all radiologically detectable disease, the patient was assessed as having achieved a complete radiological response. T-DM1 therapy has been continued for more than five years, during which the patient has maintained sustained complete radiological remission. Radiological response was assessed by serial CT imaging. Follow-up CT was performed three months after initiation of T-DM1, and subsequently at approximately six-month intervals once disease control was achieved. Complete radiological remission was defined as the disappearance of all previously identified lesions on CT imaging, consistent with Response Evaluation Criteria in Solid Tumors (RECIST)-based clinical evaluation [5]. The overall clinical course and treatment timeline are summarized in Figure 4.

Laboratory monitoring was performed every three weeks during the initial treatment phase and every six weeks after disease stabilization. Adverse events during long-term T-DM1 treatment were limited to persistent mild liver dysfunction (Table 1).

**Table 1:** Laboratory findings at five years after initiation of ado-trastuzumab emtansine (T-DM1) Laboratory data obtained five years and one month after initiation of T-DM1. Mild elevation of liver enzymes was observed without clinical symptoms. No clinically significant thrombocytopenia or hematologic toxicity was noted, and tumor markers remained stable during long-term treatment. AST: aspartate aminotransferase; ALT: alanine aminotransferase; γ-GT: gamma-glutamyl transferase; CEA: carcinoembryonic antigen; CA15-3: cancer antigen 15-3

Parameter	Patient Value	Reference Range
Complete blood cell count		
White blood cell count	8,000 /uL	3,300〜8,600/uL
Hemoglobin	13.8 g/dL	11.6〜14.8 g/dl
Platelet count	167,000/uL	158,000〜348,000/uL
Biochemistry		
Albumin	3.8g/dL	4.1〜5.1g/dL
Total bilirubin	0.9mg/dL	0.4〜1.5mg/dL
Aspartate aminotransferase	31U/L	13〜30U/L
Alanine aminotransferase	25U/L	7〜23U/L
Gamma-glutamyl transferase	64U/L	9〜32U/L
Lactate dehydrogenase	168U/L	124〜222 U/L
Tumor marker		
Carcinoembryonic antigen (CEA)	1.9ng/ml	<3.5ng/ml
Cancer antigen 15-3 (CA15-3)	17.4U/mL	<=25.0U/mL

Given the presence of underlying dyslipidemia and well-controlled diabetes mellitus, the contribution of T-DM1 to liver dysfunction could not be definitively determined. No clinically significant thrombocytopenia was observed, and the patient has maintained an excellent performance status throughout treatment.

## Discussion

T-DM1 has become a standard therapeutic option for patients with HER2-positive metastatic breast cancer, particularly in those previously treated with trastuzumab-based regimens, based on the results of pivotal clinical trials demonstrating improved survival and favorable toxicity profiles compared with conventional chemotherapy [3,4].

However, long-term complete remission under T-DM1 therapy remains rare, and reports describing sustained responses beyond several years are limited.

Recently, the concept of exceptional responders has gained increasing attention in metastatic breast cancer. A large real-world analysis identified a subset of patients who achieved exceptionally durable responses to systemic therapy, defined as responses lasting more than twice the expected progression-free survival. In that study, HER2-positive breast cancer represented the most common subtype among exceptional responders. Importantly, achievement of complete response or no evidence of disease was the strongest determinant of favorable long-term outcomes, irrespective of tumor subtype [6].

Antibody-drug conjugates, including T-DM1, have been implicated as key contributors to exceptional responses in HER2-positive metastatic breast cancer. The unique mechanism of action of T-DM1, combining targeted HER2 inhibition with intracellular delivery of a potent cytotoxic agent, may explain the depth and durability of responses observed in selected patients [7].

Resistance mechanisms to T-DM1 have been described, but their absence or delay may underlie prolonged disease control in rare cases [7]. 

Several clinical and molecular factors have been proposed as predictors of long-term response in HER2-positive metastatic breast cancer, including sustained HER2 expression and deep initial response to therapy [6,8].

In the present case, HER2 overexpression was consistently maintained at initial diagnosis and at recurrence, supporting continued sensitivity to HER2-targeted therapy.

Moreover, prior case reports have described exceptional and durable responses to T-DM1 following trastuzumab failure, and some reports have suggested a potential role of tumor microenvironmental factors in selected cases [9-11].

The present case represents an extreme example of such an exceptional response, characterized by sustained complete remission for more than five years under continuous T-DM1 therapy with minimal toxicity.

An unresolved clinical issue highlighted by this case is the optimal duration of T-DM1 therapy after achieving a complete response. Currently, there is no consensus regarding treatment discontinuation in patients with sustained remission, and most clinicians continue therapy due to concerns about relapse. Further studies are needed to identify predictive factors for exceptional responses and to establish evidence-based strategies for treatment duration in this unique patient population.

## Conclusions

This case illustrates an exceptional and durable complete response to T-DM1 in a patient with HER2-positive metastatic breast cancer. Although such long-term remissions remain rare, this case underscores the potential for sustained disease control in selected patients and highlights the need for further studies to identify predictive factors and optimize treatment duration.
